# Diagnostic performance of artificial intelligence-based computer-aided diagnosis system in longitudinal and transverse ultrasonic views for differentiating thyroid nodules

**DOI:** 10.3389/fendo.2023.1137700

**Published:** 2023-02-14

**Authors:** Lin-lin Zheng, Su-ya Ma, Ling Zhou, Cong Yu, Hai-shan Xu, Li-long Xu, Shi-yan Li

**Affiliations:** ^1^ Sir Run Run Shaw Hospital, School of Medicine, Zhejiang University, Hangzhou, China; ^2^ YinZhou No.2 Hospital, Department of Ultrasound, Ningbo, China

**Keywords:** thyroid nodule, ultrasound, artificial intelligence (AI), computer-aided diagnosis system (CAD), transverse

## Abstract

**Objective:**

To evaluate the diagnostic performance of different ultrasound sections of thyroid nodule (TN) using computer-aided diagnosis system based on artificial intelligence (AI-CADS) in predicting thyroid malignancy.

**Materials and methods:**

This is a retrospective study. From January 2019 to July 2019, patients with preoperative thyroid ultrasound data and postoperative pathological results were enrolled, which were divided into two groups: lower risk group (ACR TI-RADS 1, 2 and 3) and higher risk group (ACR TI-RADS 4 and 5). The malignant risk scores (MRS) of TNs were obtained from longitudinal and transverse sections using AI-CADS. The diagnostic performance of AI-CADS and the consistency of each US characteristic were evaluated between these sections. The receiver operating characteristic (ROC) curve and the Cohen κ-statistic were performed.

**Results:**

A total of 203 patients (45.61 ± 11.59 years, 163 female) with 221 TNs were enrolled. The area under the ROC curve (AUC) of criterion 3 [0.86 (95%CI: 0.80~0.91)] was lower than criterion 1 [0.94 (95%CI: 0.90~ 0.99)], 2 [0.93 (95%CI: 0.89~0.97)] and 4 [0.94 (95%CI: 0.90, 0.99)] significantly (P<0.001, P=0.01, P<0.001, respectively). In the higher risk group, the MRS of transverse section was higher than longitudinal section (P<0.001), and the agreement of extrathyroidal extension and shape was moderate and fair (κ =0.48, 0.31 respectively). The diagnostic agreement of other ultrasonic features was substantial or almost perfect (κ >0.60).

**Conclusion:**

The diagnostic performance of computer-aided diagnosis system based on artificial intelligence (AI-CADS) in longitudinal and transverse ultrasonic views for differentiating thyroid nodules (TN) was different, which was higher in the transverse section. It was more dependent on the section for the AI-CADS diagnosis of suspected malignant TNs.

## Introduction

Thyroid cancer is the most common type of endocrine malignancy, ranked the fifth leading cause of cancer in females worldwide (1). Fortunately, most detected thyroid nodules (TNs) are benign and have little effects on patient health (2). But for some malignant TNs, surgical resection is the first choice. Therefore, it is necessary to make differential diagnosis of TNs. Ultrasound (US) is the preferred imaging method for not only screening but also diagnosis of thyroid diseases recommended by various thyroid guidelines (3, 4). Unfortunately, subjectivity and operator dependency are the main shortcomings. This means that sonologists with different levels of experiences will have different diagnostic performance.

With the development of artificial intelligence (AI) and computer science, machine learning, especially deep learning, has shown its advantages in image classification and achieved satisfactory results (5). The application of AI based on US images for diagnosing TNs is gaining extensive attention and research (6). Many previous studies have shown that computer-aided diagnosis system based on artificial intelligence (AI-CADS) for the classification of TNs can achieve accuracy comparable to senior sonologists, reduce operator dependency, and provide a second advice in imaging diagnosis (7–10).

Previous studies have found that there was a difference in the diagnostic performance of elastography between transverse and longitudinal planes in the differentiation of benign and malignant TNs (11–13). Although there have been many literatures on the classification of TNs by AI, the difference of diagnostic efficacy between transverse and longitudinal plane remains unclear. Most of the related studies were based on a single image in transverse or longitudinal plane, and there was no comparative analysis of the different planes. Therefore, the purpose of this study was to determine whether AI-CADS would provide different results when recognizing the US images in different planes of a same TN.

## Materials and methods

### Patients and groups

**Figure 1:**
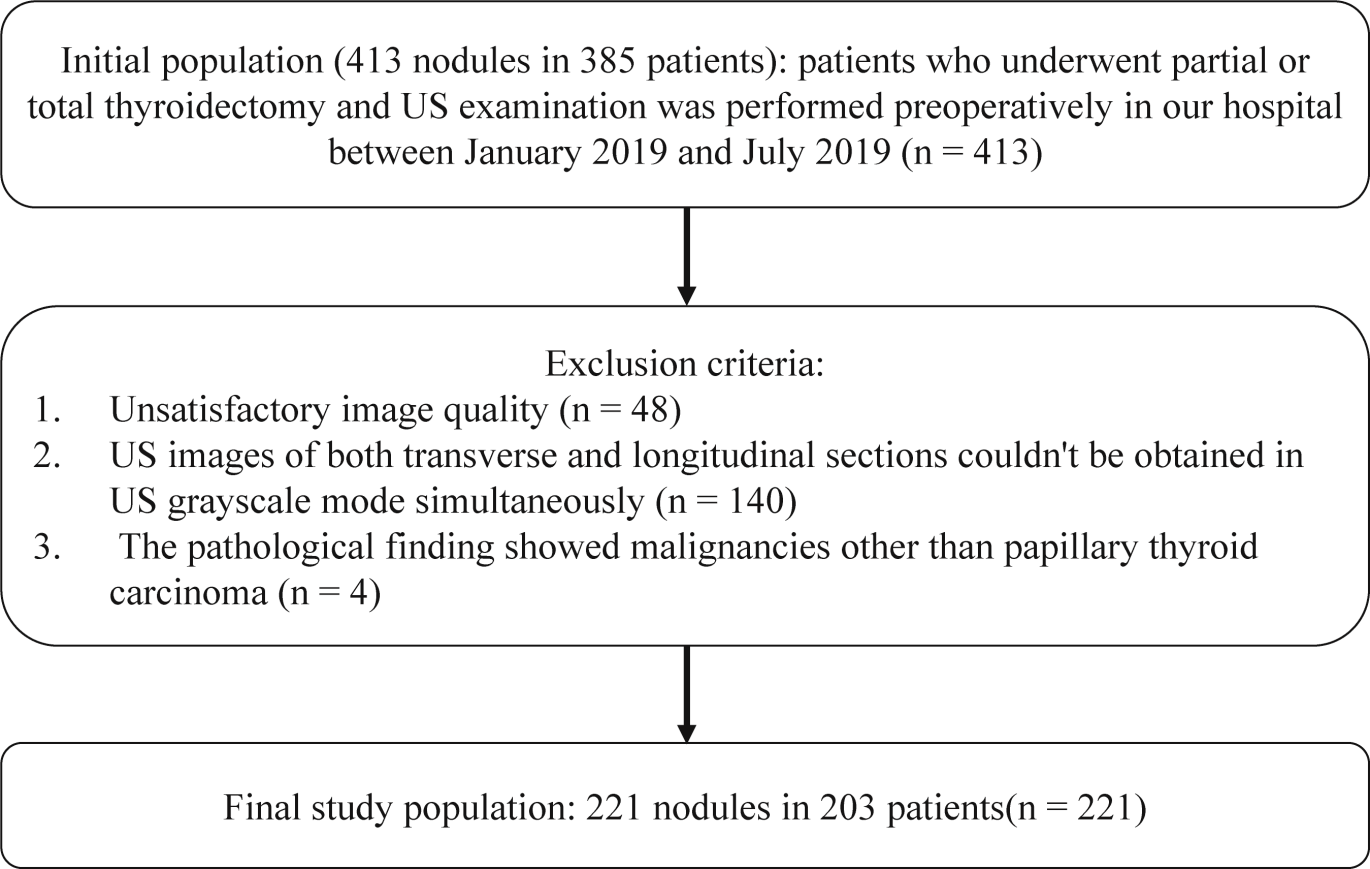
Flowchart of inclusion criteria for initial population and exclusion criteria for the final study population.

This retrospective study was approved by the Ethics Committee of our institution. Informed consent was obtained from each of the participant prior to the data collection for scientific research and potential publication of their anonymized images. The initial population included 413 nodules in 385 patients who underwent thyroidectomy in our hospital. Between January 2019 and July 2019, thyroid US examination was performed preoperatively. The inclusion criteria were as follows: (1) with clear postoperative pathological diagnosis; (2) for the malignant TNs, only papillary thyroid carcinoma were included; (3) the time interval between US and surgery was less than 3 months; (4) transverse and longitudinal planes of a same TN can be obtained in US grayscale mode. The exclusion criteria were as follows: (1) recognition for TNs was affected due to measurement calipers or other markers in US images (The TNs were covered by labels; or the measurement calipers of the TNs were larger than half or more of the minimum diameter of the TNs; and there had solid or dashed lines between the measurement calipers); (2) unsatisfactory image quality for other reasons; (3) the pathological finding showed malignancies other than papillary thyroid carcinoma. Finally, 221 TNs from 203 patients were enrolled in the study (**Figure 1**). Among them, 40 (19.70%) were male, 163 (80.30%) were female, and the age was 45.6 ± 11.6 years (range 17-73 years). According to the Thyroid Imaging Reporting and Data System of American College of Radiology (ACR TI-RADS), the enrolled TNs were divided into two groups: lower risk group (TR1, TR2 and TR3) and higher risk group (TR4 and TR5).

### US images collection

US images in transverse and longitudinal views of the TNs were acquired during the same US examination. Then the images were anonymized and randomly arranged for further analysis. The thyroid US images from multiple different devices were collected, including Philips EPIQ 5, Philips IU 22, GE LOGIQ E9, SUMSUNG RS80A, Supersonic Aixplorer and Mindray Resona 7S, with a 3-15 MHz linear transducer (L12-5, L12-5, ML6-15, L3-12, SL15-4, L14-5, respectively). The US images were obtained under optimal preset for thyroid and stored in the picture archiving and communication system, which was performed by sonologists with different experiences.

### AI-CADS analysis

The AI-CADS software (for thyroid) is developed by Demetics Medical Technology (Zhejiang, China, www.demetics-medical.com). Cascaded deep convolution neural network was used to establish this analyzing system for automatic detection and identification of benign and malignant TNs (14).

**Figure 2:**
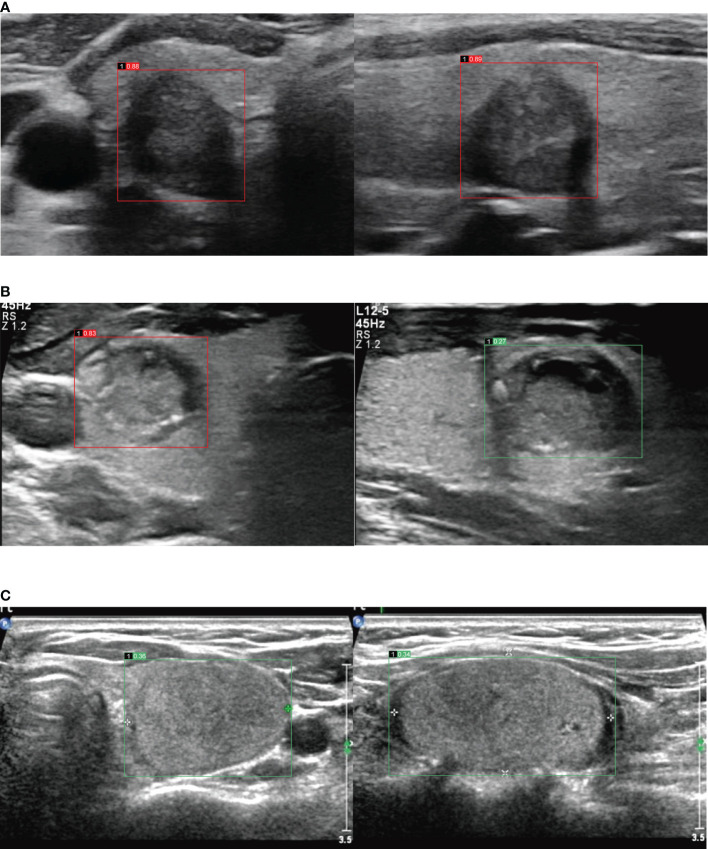
**(A)** Images in a 60-year-old woman with papillary thyroid carcinoma in the right lobe of thyroid, image of transverse section (the left), malignant risk score is 0.88, suggests malignant, image of longitudinal section (the right), malignant risk score is 0.89, suggests malignant. **(B)** Images in a 34-year-old man with papillary thyroid carcinoma in the right lobe of thyroid, image of transverse section (the left), malignant risk score is 0.83, suggests malignant, image of longitudinal section (the right), malignant risk score is 0.27, suggests benign. **(C)** Images in a 57-year-old woman with follicular thyroid adenoma in the left lobe of thyroid, image of transverse section (the left), malignant risk score is 0.36, suggests benign, image of longitudinal section (the right), malignant risk score is 0.34, suggests benign.

Images with DICOM or jpg format were inputted in the AI-CADS, and TNs in US images could be detected automatically and assessed simultaneously. The result was demonstrated by using malignant risk score (MRS), which ranges from 0 to 1. With the increased MRS, the malignant risk of TNs increases relatively (**Figure 2**). MRS of each TN was compared between transverse and longitudinal plane. This comparison was performed in the lower risk group, higher risk group and all of the enrolled TNs, respectively. Furthermore, for comparing the diagnostic efficacy of AI-CADS for differentiating TNs in transverse and longitudinal planes, the diagnostic criteria were designed to the following four categories:

Criterion 1, the maximum MRS between transverse and longitudinal planes indicates malignant (MRS larger than cutoff value);

Criterion 2, the MRS in the transverse plane indicates malignant, regardless of the MRS in the longitudinal plane;

Criterion 3, the MRS in the longitudinal plane indicates malignant, regardless of the MRS in the transverse plane;

Criterion 4, the average MRS of the transverse and the longitudinal plane indicates malignant.

### US characteristics analysis manually

The anonymized and randomly arranged images were evaluated by two sonologists with more than 10 years of experience independently. For each enrolled TN, the US characteristics, including size, composition, echogenicity, shape, margin, calcification and extrathyroidal extension were evaluated. If no consensus was reached, arbitration from another sonologist (with more than 20 years of experience) was performed. The three reviewers were blinded to the findings of each other, the pathological results and other clinical information of the TNs. The ACR TI-RADS category was arranged according to the total score of these information (4). These US characteristics were compared between transverse and longitudinal plane in different two groups.

### Statistical analysis

SPSS 22.0 statistical software and R version 3.5.3 were used to analyze the data. If the variables were quantitative and normal, the mean ± standard deviation was used for statistical description, and the median with interquartile range (IQR) was used for the non-normal variables. Counting data was presented as numbers and percentage. Difference in MRS between transverse and longitudinal planes of TNs were analyzed by Wilcoxon signed-rank test for paired samples. The receiver operating characteristic (ROC) curve was drawn according to the pathological results as the gold standard to find the best diagnostic cut-off value. Sensitivity, specificity, positive predictive value (PPV), negative predictive value (NPV), diagnostic accuracy, false-negative rate (FNR) and false positive rate (FPR) were calculated. In addition, ROC curve was used to compare the diagnostic efficacy among different diagnostic criteria of AI-CADS. The area under ROC curve (AUC) was compared by Z test. The Cohen *κ*-statistic was used to assess the agreement of each US characteristic between the two planes. The degree of agreement was defined as follows: 0.00-0.20, slight agreement; 0.21-0.40, fair agreement; 0.41-0.60, moderate agreement; 0.61-0.80, substantial agreement; 0.80-1.00, almost perfect agreement. McNemar’s test was used to compare proportions of each US characteristic between the two planes. *P*<0.05 was considered as statistically significant.

## Results

Totally, 221 TNs were enrolled and confirmed by postoperative pathology, including 166 cases of malignant TNs and 55 cases of benign TNs. The main reasons for operating on these benign TNs were: (1) complicated with thyroid carcinoma: the patients had both malignant and benign nodules in the thyroid lobe; (2) the fine-needle aspiration reported follicular tumor before operation; (3) local compressive symptoms of TNs. For the malignant TNs, only papillary thyroid carcinoma was included, the average size of malignant TNs was 1.28 ± 0.82 cm (range 0.32-5.61 cm). The benign TNs included nodular goiter (n=33), follicular thyroid adenoma (n=8) and nodular goiter with follicular adenoma (n=14), and the average size of them was 3.14 ± 1.30 cm (range 0.55-5.67 cm). According to the ACR TI-RADS classification, 43 nodules were arranged in the lower risk group (TR2 and TR3), and 178 nodules were in higher risk group (TR4 and TR5).

### Comparison of diagnostic performance of AI-CADS for differentiating TNs in transverse and longitudinal planes

**Figure 3:**
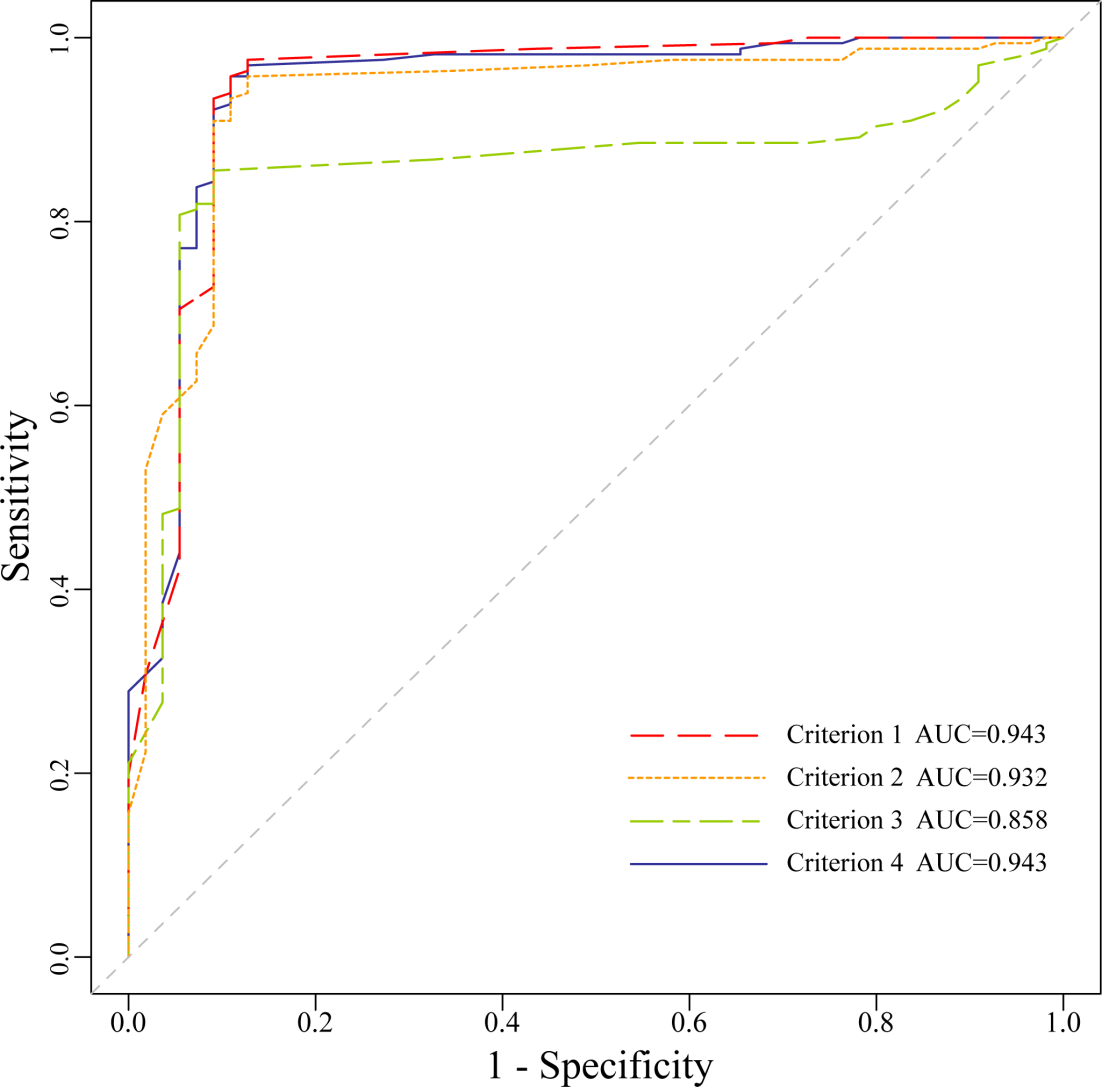
The Receiver operating characteristic curves of malignant risk score for the four criteria.

ROC curves of MRS with different four criteria were drawn (**Figure 3**). The cutoff value of criterion 1 was 0.53, and 0.40, 0.41 and 0.48 for criterion 2, 3, 4 respectively. The sensitivity, specificity, PPV, NPV, FNR, FPR, accuracy and AUC value of each criterion were displayed in **Table 1**. The AUC value of criterion 3 was lower than criterion 1, 2 and 4 respectively (*P*<0.05). The AUC value of criterion 4 was the highest one without significant difference compared with criterion 1 or criterion 2 (*P*>0.05).

**Table 1:** Performance of AI-CADS in transverse and longitudinal planes for differentiating TNs.

Criterion	SEN (%)	SPE (%)	PPV (%)	NPV (%)	FNR (%)	FPR (%)	ACC (%)	AUC (95% CI)	*P* Value
1	159/166 (95.78)	49/55 (89.09)	159/165 (96.36)	49/56 (87.50)	7/166 (4.22)	6/55 (10.91)	208/221 (94.12)	0.943 (0.90-0.99)	0.33 ^a^ <0.001 ^b^ 0.99 ^c^
2	159/166 (95.78)	48/55 (87.27)	159/166 (95.78)	48/55 (87.27)	7/166 (4.22)	7/55 (12.73)	207/221 (93.67)	0.932 (0.89-0.97)	0.01 ^b^ 0.37 ^c^
3	142/166 (85.54)	50/55 (90.91)	142/147 (96.60)	50/74 (67.57)	24/166 14.46	5/55 (9.09)	172/221 (86.22)	0.858 (0.80-0.91)	<0.001 ^c^
4	159/166 (95.78)	49/55 (89.09)	159/165 (96.36)	49/56 (87.50)	7/166 (4.22)	6/55 (10.91)	208/221 (94.12)	0.943 (0.91-0.99)	–

AI-CADS, computer-aided diagnosis system based on artificial intelligence; TN, thyroid nodule; SEN, sensitivity; SPE, specificity; PPV, positive predictive value; NPV, negative predictive value; FNR, false negative rate; FPR, false positive rate; ACC, accuracy; AUC, area under receiver operating characteristic curve; CI, confidence intervals.

Criterion 1, the maximum MRS between transverse and longitudinal planes indicates malignant.

Criterion 2, the MRS in the transverse plane indicates malignant, regardless of the MRS in the longitudinal plane.

Criterion 3, the MRS in the longitudinal plane indicates malignant, regardless of the MRS in the transverse plane.

Criterion 4, the average MRS of the transverse plane and the longitudinal plane indicates malignant.

a P value was from AUC comparison with criterion 2.

b P value was from AUC comparison with criterion 3.

c P value was from AUC comparison with criterion 4.

### Differences of MRS in transverse and longitudinal planes of the same TNs

Overall, MRS of TNs in transverse and longitudinal planes obtained by AI-CADS was 0.80 (IQR: 0.46) and 0.77 (IQR: 0.47) respectively. There was a significant difference between them (*P*=0.001). There was no significant difference of MRS between the two planes in the lower risk group (*P*=0.70). For the higher risk group, the MRS of transverse planes was higher than that of longitudinal planes (*P*<0.001) (**Table 2**).

**Table 2:** Difference of MRS in transverse and longitudinal planes of TNs.

MRS	Transverse plane (median/IQR)	Longitudinal plane (median/IQR)	*P* Value
Total (n=221)	0.80/0.46	0.77/0.47	0.001
Lower risk group (n=43)	0.38/0.03	0.38/0.03	0.70
Higher risk group (n=178)	0.83/0.11	0.79/0.16	<0.001

MRS, malignant risk scores; TN, thyroid nodule; IQR, interquartile range.

### Agreement of US characteristics in transverse and longitudinal planes

In the higher risk group, the agreement of extrathyroidal extension and shape was moderate and fair, with the *κ* value of 0.48 and 0.31 respectively. The agreement of other US features including size, composition, echogenicity, calcification, and margin (excluded extrathyroidal extension) was substantial or almost perfect, the *κ* values were 0.81, 0.85, 0.96, 0.84 and 0.67, respectively. In the lower risk group, the agreement of all of these US features was substantial or almost perfect. In the higher risk group, the ratio of a taller-than-wide shape of TNs in transverse plane (130/178, 73.03%) was higher than that in longitudinal plane (65/178, 36.52%, *P*<0.001). Furthermore, there were 18.54% (33/178) of TNs which presented extrathyroidal invasion in transverse plane, and this ratio was also higher than that in longitudinal plane (21/178, 11.80%, *P*=0.02) (**Table 3**).

**Table 3:** Agreement of US characteristics in transverse and longitudinal planes.

Features	Higher risk group (n=178)			Lower risk group (n=43)		
	Agreement ^a^ (n)	Disagreement (n)	*κ*	Agreement ^a^ (n)	Disagreement (n)	*κ* ^b^
Size (<1cm vs ≥1cm)	161	17	0.81	43	0	–
Composition ^c^	176	2	0.85	39	4	0.82
Echogenicity ^d^	175	3	0.96	43	0	1.0
Shape (with or without taller-than-wide)	109	69	0.31	43	0	–
Margin ^e^	149	29	0.67	43	0	–
Calcification ^f^	166	12	0.84	41	2	0.65
Extrathyroidal Extension (without or obvious invasion)	154	24	0.48	43	0	–

a , Agreement indicated the ultrasound features of the nodules in transverse and longitudinal plane was uniform.

b , No statistics were computed because variables were constants, so κ values were missing.

c , The composition of nodules was classified into the following three categories: cystic or almost cystic or spongiform, mixed cystic and solid and solid or almost solid.

d , The echogenicity of nodules was classified into the following four categories: anechoic, hyperechoic or isoechoic, hypoechoic and very hypoechoic.

e , The margin of nodules was classified into the following two categories: smooth or ill-defined, lobulated or irregular.

f , The calcification of nodules was classified into the following four categories: none or large comet-tail artifacts, macrocalcifications, peripheral (rim) calcifications and punctate echogenic foci.

## Discussion

The performance of AI-CADS in longitudinal and transverse ultrasonic views for differentiating TNs was investigated in this study. The results showed that the diagnostic efficacy of different criteria was different. The AUC in the longitudinal plane was lower than that of other criteria (*P*<0.05). The MRS obtained from AI-CADS was different between longitudinal and transverse views not only for the overall enrolled TNs but also in the higher risk group (TR4 and TR5). The agreement of extrathyroidal extension and shape between the two planes was relative lower in the higher risk group than that of other US characteristics or in the lower risk group, which may be the main reason leading to the results. These findings indicated that the plane selected for AI-CADS could influence the diagnostic performance for differentiating TNs, especially for the TNs with higher risk of malignancy. It is not appropriate to use only the longitudinal views as the analysis image for AI-CADS.

Similar findings on elastography and the taller-than-wide shape in TNs have been published previously, when compared the diagnostic performance between longitudinal and transverse planes (11–13, 15). These indicate that any US technique requires a standard image acquisition protocol. AI-CADS based on US could be used to reduce the subjectivity to a certain extent. The AI-CADS with the ability of real-time evaluation is undoubtedly a good diagnostic aid for sonologists. At present, there are many AI-CADS software for the diagnosis of TNs, and have a high accuracy, some even can reach the diagnostic level of senior sonologists (7–9, 16). However, there is no related research on the diagnostic results of different planes of the AI-CADS and detailed explanation on how to standardize the use of this technique (17). As a three-dimensional structure of TNs, the diagnostic information of different planes of a same TN are different. If the diagnostic results of a TN from different planes using the AI-CADS are different, or even completely opposite, it will bring confusion to sonologists. Therefore, it is necessary to compare the diagnostic performance of AI-CADS for differentiating TNs in different planes and to analyze the possible reasons for the difference.

In this study, the diagnostic efficacy of four different criteria were compared each other and the results showed that the AUC value of criterion 3 was the lowest (AUC=0.86), which was different from that of criterion 1, criterion 2 and criterion 4 (AUC=0.94, 0.93, 0.94, *P*<0.05) respectively, and there was no significant difference among the latter three.

Then, the differences of MRS between the transverse and longitudinal planes were further analyzed. The MRS of the transverse plane was higher than that of the longitudinal plane not only for the overall enrolled TNs (*P*=0.001) but also in the higher risk group (*P*<0.001). There was no significant difference between the two planes in the lower risk group (*P*=0.70). This may be attributable to the nature of the benign TNs. No matter what plane is used, there are no malignant signs for the benign TNs. Therefore, AI-CADS have a relatively low dependence on the plane. However, for moderately or highly suspicious TNs, different planes have different malignant signs and diagnostic information. When AI-CADS is used, the dependency of the plane may be increased markedly. The results of this study showed that a better ability to identify malignant signs may be appeared in the transverse plane.

A challenge of deep learning is the inexplicability of the results. For a “black box” result, it is not clear which image features are used to classify (18, 19). However, according to previous studies, some US features, such as composition, echogenicity, shape, margin, calcification and extrathyroidal extension, are the main basis for distinguishing benign and malignant TNs, among which microcalcification, irregular margin (including extrathyroidal invasion) and taller-than-wide shape are the three most specific features of thyroid carcinoma (3, 4, 20). Some classification systems based on AI analyzed and weighted these features to identify TNs (21, 22). In this study, the agreement of US features of TNs between transverse and longitudinal planes were analyzed. The agreement of extrathyroidal invasion and shape of TNs in higher risk group were moderate and fair (*κ*=0.48, 0.31 respectively). There were 73.03% (130/178) of TNs in the higher risk group which presented a taller-than-wide shape in transverse plane, and this ratio was higher than that in longitudinal plane (65/178, 36.52%) (*P*<0.001). Furthermore, the proportion of extrathyroidal invasion in transverse plane (33/178, 18.54%) was also higher than that in longitudinal plane (21/178, 11.80%) (*P*=0.02).

**Figure 4:**
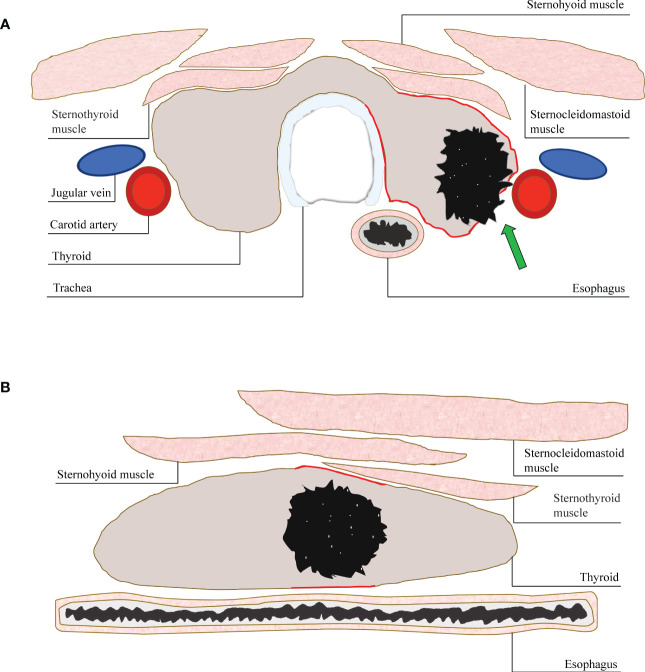
**(A)** A diagram of displaying extrathyroidal invasion in transverse and longitudinal sections. The anterior, posterior, medial and lateral margin (red line) of thyroid could be displayed simultaneously in the transverse section. The invasion (arrow) could be assessed more accurately. **(B)** While, only the anterior and posterior margin (red line) could be shown in the longitudinal section. The invasion of medial or lateral margin could be missed.

ACR TI-RADS committee pointed out that a taller-than-wide shape should be evaluated in the transverse plane. In the past study, AUC value of nodules with a taller-than-wide shape in transverse plane was higher than that in longitudinal plane (15). This result is similar to present study. Obvious extrathyroidal invasion is a reliable feature that directly indicates that the nodule is malignant (23). TN is a three-dimensional structure, and the relationship between the nodule and the thyroid border reflected on the transverse and longitudinal plane is different. In the transverse plane, it can reflect the invasion to the anterior, posterior, medial and lateral margin of the thyroid, especially to the trachea on the medial and the blood vessels on the lateral. While in the longitudinal plane, it mostly reflects the invasion to the anterior and posterior edge of the thyroid. Therefore, this may be the possible cause that the extrathyroidal invasion of TNs can be shown to a greater extent in the transverse plane (**Figure 4**).

This study had several limitations. Firstly, the study was a retrospective study, the images used were not prospectively collected according to a norm. Secondly, because the standard used in this study was the pathological findings after the surgical resection, it did not include a large number of benign nodules, and there were only 3 cases of benign nodules less than 1cm. The overall diagnostic ability of AI-CADS for small nodules should be further analyzed. Thirdly, only malignant TNs with papillary carcinoma were enrolled in this study. TNs with other types of thyroid cancer were excluded. Because the AI-CADS used in this study was established based on a training set of US images mainly of thyroid papillary carcinoma (which has the highest incidence of thyroid malignant tumors). There is relatively little training for other types of thyroid cancer, similar to previous studies (10, 24). This will lead to poor diagnostic ability of nodules in other pathological type by using AI-CADS. Although only thyroid papillary carcinoma was included in this study, it still has significant clinical implication because of its high proportion in thyroid cancer. Further study of AI-CADS focused on other pathological type of thyroid cancer should be designed.

In summary, the diagnostic performance of AI-CADS in longitudinal and transverse ultrasonic views for differentiating TNs was different, which was higher in the transverse plane than in the longitudinal plane. It was more dependent on the plane for the AI-CADS diagnosis of suspected malignant TNs.

## Data availability statement

The original contributions presented in the study are included in the article/supplementary material. Further inquiries can be directed to the corresponding author.

## Ethics statement

The studies involving human participants were reviewed and approved by Sir Run Run Shaw Hospital, Zhejiang University, School of Medicine. Written informed consent for participation was not required for this study in accordance with the national legislation and the institutional requirements.

## Author contributions

L-LZ wrote the manuscript. L-LZ, S-YM and S-YL contributions to concept and design of the study. LZ and CY collected data. S-YM, H-SX and L-LX analyzed the data. S-YL made suggestions for some of the work. S-YM and S-YL revised the work. All authors contributed to the article and approved the submitted version.
